# Supplementary material for: Cost-effectiveness of diagnostic algorithms including lateral-flow urine lipoarabinomannan for HIV-positive patients with symptoms of tuberculosis
Source: PLoS One. 2020 Jan 30;15(1):e0227138. doi: 10.1371/journal.pone.0227138 (PMC6992347; doi:10.1371/journal.pone.0227138)
Supplement: Attachment — Submitted filename: Response to Reviewers.docx [file pone.0227138.s002.docx]

**Response to Reviewers**

**PONE-D-19-20635**

**Cost-effectiveness of diagnostic algorithms including lateral-flow urine lipoarabinomannan for HIV-positive patients with symptoms of tuberculosis**

**Journal Requirements:**

1. When submitting your revision, we need you to address these additional requirements.

Please ensure that your manuscript meets PLOS ONE's style requirements, including those for file naming. The PLOS ONE style templates can be found at:

http://www.journals.plos.org/plosone/s/file?id=wjVg/PLOSOne_formatting_sample_main_body.pdf and <http://www.journals.plos.org/plosone/s/file?id=ba62/PLOSOne_formatting_sample_title_authors_affiliations.pdf>

**Réponse : we modified according to the requirements of PLOS ONE :**

- The affiliation of the authors and we wrote a legend about the work of each author

- We modified the presentation of figures and tables titles and legends

2. We noticed you have some minor occurrence(s) of overlapping text with the following previous publication(s), which needs to be addressed: :

<https://doi.org/10.5588/ijtld.13.0630>

<https://doi.org/10.1371/journal.pone.0170976>

<https://doi.org/10.1371/journal.pmed.1002792>

<https://dx.doi.org/10.1371%2Fjournal.pone.0117009>

https://halshs.archives-ouvertes.fr/halshs-01241824/file/2015.34.pdf

In your revision ensure you cite all your sources (including your own works), and quote or rephrase any duplicated text outside the Methods section. Further consideration is dependent on these concerns being addressed.

**Response:** Thank you, we modify as follows:

- <https://doi.org/10.5588/ijtld.13.0630> 🡺 This reference has been added [19] (lines 148, 157, 174 of the manuscript) and we modify the passages where there were certain occurrences (lines 148 to 154 and 179 to 182 of the manuscript)
- <https://doi.org/10.1371/journal.pone.0170976> 🡺This reference [15] has been added lines 78 and 110 of the manuscript
- ttps://halshs.archives-ouvertes.fr/halshs-01241824/file/2015.34.pdf 🡺 we modify the sections where there were certain occurrences (lines 355 to 357 of the manuscript)
- <https://dx.doi.org/10.1371%2Fjournal.pone.0117009>🡺This reference [30] has been added lines 206

3. We note that you have numbered 5 affiliations in your title page, but only assigned 1,2,3, & 4 to authors.  Please assign all the affiliations listed to an author, or remove no.5 if it is not needed

**Response:** Thank you, the mistake has been corrected.

4. We note that you have stated that you will provide repository information for your data at acceptance. Should your manuscript be accepted for publication, we will hold it until you provide the relevant accession numbers or DOIs necessary to access your data. If you wish to make changes to your Data Availability statement, please describe these changes in your cover letter and we will update your Data Availability statement to reflect the information you provide.

**Response:** thanks for the comment, it is well noted

**We have added a paragraph “supporting information” line 505-507**

**5. Review Comments to the Author**

**Reviewer #1:**

1. HIV patients often have difficulties to produce sputum, thus only the GeneXpert, LF-LAM seem to be fast and effective ways to detect TB. What the purpose of the research? What the novelty of the research?

***Response***:

Thank you for this question. To enlighten the reader we have added a paragraph on the purpose as well as the novelty of this research at the end of the introduction (lines 85 to 88 of the manuscript)

2. All the figures in the manuscript were not clear, need to revise.

***Response***: Thank you for this remark. We have improved the quality of the figures. In case our article is accepted, we will also discuss with PLOS One production team how to improve further the quality of the figures.

**---------------------------------------------------------------------------------------------**

**Reviewer #2:** Manuscript PONE-D-19-20635: Cost-effectiveness of diagnostic algorithms including lateral-flow urine lipoarabinomannan for HIV-positive patients with symptoms of tuberculosis

- Introduction: This paper focuses on the LF-LAM assay, however, the assay is not described anywhere in the manuscript. Please consider adding a brief overview of the assay to familiarize the reader with what it does.

***Response***: Thank you for this proposition, we have added a brief overview of the assay (lines 62 to 71 of the manuscript)

- Line 110: The title of your figure should give the reader a brief description of what they are looking at. Please consider revising this title as well as those in other figures and tables in the manuscript as many are very sparse in description.

***Response***: Thank you for this remark, we agree. We have changed Figure titles as follows:

- **Fig 1. Diagnostic algorithms using microscopy or GeneXpert alone or in combination with chest X-ray versus LF-LAM alone or in addition to other TB diagnostic tools** (lines 125-126 of the manuscript)
- **Fig 2. Cost-effectiveness planes** (This graph represent the differences in costs and DALYs between algorithms. DALYs are plot on the x axis and costs on the y axis. Results in costs and differences in DALYs averted observed in the 10, 000 bootstrap replicates) lines 296 to 298 of the manuscript)
- **Fig 3. Probability of cost-effectiveness of each algorithm according to the society’s Willingness To Pay (WTP) (€2,673) (**lines 308-309 of the manuscript)

- Cost estimation: Line 129: The acronym IPSOR is used without definition. Please define.

***Response*** : Thank you for this remark, the acronym has been defined **(**lines 145-146 of the manuscript)

- Cost estimation: Line 163: The acronym CXR is used without definition. Please define.

***Response*** : Thank you for this remark, we have changed CXR by Chest X-ray. **(**line 180 of the manuscript)

- Cost estimation: Line 155: “pipette” should be plural in this case, should be “pipettes”

***Response*** :Thank you for this remark, we have added an « s » to the word pipettes **(**line 172 of the manuscript)

- Cost estimation: Line 164: Here and in several places in the manuscript you indicate that a 4% discount was applied, however, I could not find the reasoning for this discount. Please consider adding an explanation to the manuscript.

***Response*** :. Thank you for this comment. We have added an explanation and justified the rate discount chosen which is a recommendation of the French Health Authority **(**lines 181-182 and 189 of the manuscript)

- Line 173: “Tables 1” does not need to be plural in this instance, should be “Table 1”

***Response***: Thank you , we have deleted the « s » **(**line 192 of the manuscript)

- Study population: Line 199: Are these all of the statistics that are available for the patients? Consider adding additional statistics if possible.

***Response***: Thank you. We have included some additional description of the population as suggested **(**lines 218 to 225 of the manuscript)

- Study population: Line 200: “patients had a confirmed TB” should not have an “a”. Should read “patients had confirmed TB”

***Response*** : Thank you , we have deleted the « a » **(**line 226 of the manuscript)

- Line 218: “Tables 3” does not need to be plural. Should read “Table 3”

***Response*** : Thank you , we have deleted the « s » **(**line 244 of the manuscript)

- Line 249: “Figures 2” does not need to be plural. Should read “Figure 2”

***Response*** : Thank you, we have removed the « s » **(**line 275 of the manuscript)

- Line 256: The acronym WTP is used without definition. Please define.

***Response*** : Thank you, we have removed the acronym all over the manuscript and used the full word and, we propose to add a definition at this level **(**lines 202-203 of the manuscript)**.**

- Lines 274-279: This paragraph is very confusing the way that it is written, please consider revising.

***Response:*** Thank you for this comment. We have modified the paragraph as suggested **(**line 299 to 306 of the manuscript)

- Line 326: The acronym GNI is used without definition. Please define.

***Response:*** Thank you for this comment. Thank you, we have removed the acronym all over the manuscript and used the full word.
