# Supplementary material for: Cost-effectiveness of diagnostic algorithms including lateral-flow urine lipoarabinomannan for HIV-positive patients with symptoms of tuberculosis
Source: PLoS One. 2020 Jan 30;15(1):e0227138. doi: 10.1371/journal.pone.0227138 (PMC6992347; doi:10.1371/journal.pone.0227138)
Supplement: Attachment — Submitted filename: Response to Reviewers_.docx [file pone.0227138.s003.docx]

**Response to Reviewers**

**PONE-D-19-20635R1
Cost-effectiveness of diagnostic algorithms including lateral-flow urine lipoarabinomannan for HIV-positive patients with symptoms of tuberculosis
PLOS ONE**

 **6. Review Comments to the Author**

Please use the space provided to explain your answers to the questions above. You may also include additional comments for the author, including concerns about dual publication, research ethics, or publication ethics. (Please upload your review as an attachment if it exceeds 20,000 characters)

Reviewer #1: The revised manuscrip covered all my comments, while there are still exist several minimal mistakes in the revised mauscript.

minimal comments:

1. In line 152, there are two dots, remove one.

***Response***: Thank you , we have deleted the dots **(**line 152 of the manuscript)

2. In the revise manuscript, several references "underline", remove the underline. such as, in line 181, 185, 189, et al.

***Response*** : Thank you for this remark, we have modified all “underline” references

3. In line 156, reference [17], [19], should be changed into [17, 19].

***Response*** : Thank you for this remark, we have changed reference [17] [19] by [17,19] **(**line 156 of the manuscript)

4. In line 158, in the sentence “number of patients/day", should be changed into "number of patients per day".

***Response*** : Thank you for this remark, we have changed “number of patients/day” by “number of patients per day” **(**line 158 of the manuscript)

5. In line 160, "First" should be changed into "Firstly".

***Response*** : Thank you for this remark, we have changed “First” by “Firstly” **(**line 160 of the manuscript)

6. In Table 1, in the setences "Age at oneset of disablity a" and "Age of death a", what the word a stand for?

***Response***: Thank you for this careful reading, this is a mistake, we have deleted the “a” **(table 1 of the manuscript)**

7. In the line 201, the reference [23-24], shoukd be changed into [23, 24].

***Response*** : Thank you for this remark, we have changed reference [23-24] by [23, 24] **(**line 199 of the manuscript)

8. In the line 213, the reference [17-18], shoukd be changed into [17, 18].

***Response*** : Thank you for this remark, we have changed reference [17-18] by [17, 18] **(**line 211 of the manuscript)

9. In the line 222, 100~199 cells/u, should be changed into 100~199 cells/uL.

***Response*** : Thank you for this remark, we have changed 100~199 cells/u by 100~199 cells/uL **(**line 220 of the manuscript)

10. The references in this manuscript should be adjust to fit the PloS One. I saw many differences in the references, such as reference 10, 201-9, should be changed into 201-209. Please doubcheck the references.

***Response*** : Thank you for this remark, we have adjusted the following refeneces : 6, 10, 23, 28, 35, 39, 40, 42

Reviewer #2: None. The authors have addressed all of the suggestions that were put forth by me in the initial review. Thank you.

7. PLOS authors have the option to publish the peer review history of their article ([what does this mean?](https://journals.plos.org/plosone/s/editorial-and-peer-review-process#loc-peer-review-history)). If published, this will include your full peer review and any attached files.

If you choose “no”, your identity will remain anonymous but your review may still be made public.

***Response*** : Thank you, this is well noted
